# Adsorption of Oxytetracycline Hydrochloride by Iron-Doped Sodium Alginate Gel Composite Biochar Microspheres: Performance and Mechanism

**DOI:** 10.3390/gels12050360

**Published:** 2026-04-26

**Authors:** Rong Chen, Jianlin Zhou, Weiyin Liu, Renjian Deng, Lingling Wang, Xin Lu, Zhang Chen, Guoliang Chen, Zhixian Li

**Affiliations:** 1 College of Resource Environment and Safety Engineering, Hunan University of Science and Technology, Xiangtan 411201, China; 23020101004@mail.hnust.edu.cn (R.C.); deng800912@163.com (R.D.); w21390521@163.com (L.W.); lx15670212021@163.com (X.L.); chengroup_science@163.com (Z.C.); glchen@hnust.edu.cn (G.C.); zhixianlimao@163.com (Z.L.); 2 Hunan Province Key Laboratory of Clean Utilization of Coal Resources and Mine Environmental Protection, Hunan University of Science and Technology, Xiangtan 411201, China; 3 School of Chemistry and Chemical Engineering, Hunan University of Science and Technology, Xiangtan 411201, China; 1061001@hnust.edu.cn

**Keywords:** Sodium alginate, biochar microspheres, oxytetracycline hydrochloride, oxidative modification, adsorption models, microsphere formation

## Abstract

Conventional powdered biochar encounters severe bottlenecks in practical water treatment, such as difficult separation, easy loss, and potential secondary pollution. This work aimed to develop recyclable and high-performance adsorbents by preparing iron-doped biochar/sodium alginate composite microspheres (BC/MBC500-ALF) through Fe^3+^ cross-linking. Using corn stalk biochar and KMnO_4_-modified biochar as adsorbent components and sodium alginate (SA) as a green shaping matrix, SA formed a stable egg-box hydrogel network to convert powdered biochar into uniform microspheres. Batch adsorption experiments revealed that the optimal pH for oxytetracycline (OTC) adsorption was 9, with adsorption capacities of 136.28 mg/g for BC500-ALF and 182.91 mg/g for MBC500-ALF. Kinetic analysis showed that BC500-ALF followed pseudo-first-order kinetics (*R*^2^ = 0.983) dominated by physisorption, while MBC500-ALF fitted pseudo-second-order kinetics (*R*^2^ = 0.994) dominated by chemisorption. The maximum Langmuir adsorption capacities at 308 K were 220.75 mg/g and 495.05 mg/g, respectively. Thermodynamic parameters confirmed a spontaneous and endothermic process. The adsorption mechanisms involved hydrogen bonding, π–π stacking, electrostatic attraction, metal-bridging complexation, and Fe–Mn oxide-mediated redox reactions. SA exerted dual functions in structure stabilization and adsorption enhancement. This composite provides an efficient and eco-friendly approach for tetracycline antibiotic pollution control in aqueous environments.

## 1. Introduction

Oxytetracycline hydrochloride (OTC), a typical tetracycline (TC) antibiotic, is a broad-spectrum agent widely used in medicine, animal husbandry, and aquaculture [1]. However, owing to its stable chemical structure, strong persistence, incomplete absorption in animals and humans, and poor biodegradability, OTC tends to accumulate continuously in the environment, posing potential risks to human health and even the entire ecosystem. Previous studies have demonstrated that only 20–30% of the administered OTC can reach target organisms in aquaculture practices, while approximately 75% of antibiotic residues are released into aquatic ecosystems [2]. Such extensive and unregulated application exerts intense selective pressure on microbial communities and accelerates the emergence of multidrug-resistant phenotypes via horizontal gene transfer. Although OTC usually exists at trace concentrations, its inherent chemical stability and the bioaccumulation potential of its toxic metabolites make it a persistent and significant threat to aquatic ecosystems and human health [3]. Therefore, the development of cost-effective, efficient, and eco-friendly technologies for the remediation of antibiotic contamination has become an important research direction in the field of environmental science.

At present, common pollutant removal technologies include advanced oxidation processes [4], biological treatment [5] and adsorption techniques [6]. Biochar is a carbon-rich polymer produced by the pyrolysis of carbonaceous biomass under anaerobic or oxygen-limited conditions. As the world’s largest producer of crop straw, China possesses enormous potential in biomass resources [7]. Corn straw accounts for the dominant fraction of crop straw and is produced in large quantities. Corn straw is mainly composed of cellulose, hemicellulose, and lignin, with a rich carbon skeleton, making it an ideal precursor for biochar preparation [8]. It features high porosity, surface area, adsorption capacity, and cation exchange capacity, and has been widely applied in agriculture, energy storage, and environmental management [9]. To further improve the removal efficiency of biochar, researchers have adopted various modification strategies to optimize its surface functional group composition, pore structure, and adsorption selectivity. Fan et al. fabricated corncob-derived modified biochar (OBC) by combining biosynthesized iron oxide nanoparticles from *Aquabacterium* sp. XL4 and carbon nanotubes, followed by pyrolysis at 600 °C for 4 h; the optimized material had a large specific surface area of 237.51 m^2^/g and a maximum adsorption capacity of 72.59 mg/g for OTC [10]. Cui et al. prepared a magnetic composite adsorbent by modifying mango lignocellulosic kernel biochar with MnFe_2_O_4_ and Cu@Zn-BDC MOF. The composite exhibited a maximum tetracycline adsorption capacity of 42.67 mg/g, 58% higher than pristine biochar, and could effectively remove tetracycline from aqueous solution and hospital wastewater [11].

Against the background of “dual-carbon” goals (carbon peaking and carbon neutrality), adsorption, characterized by low cost, high efficiency, easy operation and environmental friendliness, is regarded as a green and low-carbon technology for the treatment of emerging contaminants, and has been widely applied in the remediation of OTC pollution in aqueous solutions [12]. Among various adsorbents, biochar has attracted considerable attention from researchers due to its wide availability, low cost, high porosity, and abundant oxygen-containing functional groups (e.g., carboxyl, hydroxyl, methoxyl and phenolic groups), which enhance the binding capacity of biochar with other substances [13]. However, conventional powdered biochar suffers from several application drawbacks: difficult separation and recovery, easy loss and secondary pollution, high bed pressure drop, easy clogging, and inconvenient regeneration and reuse. This results in powdered biochar often being regarded as a disposable consumable, which is inconsistent with the principles of circular economy and sustainable development. To overcome the shortcomings of powdered biochar and endow it with excellent engineering applicability for convenient use in practical water treatment facilities, biochar shaping technology has emerged.

Common shaping processes include granulation [14], extrusion molding [15], immobilization [16,17], etc. Briens et al. systematically compared the shaping effects of three liquid binders (hydroxylpropyl methylcellulose (HPMC), molasses, ammonium nitrate) on biochar powder during drum granulation. It was found that different binders influenced the granulation mechanism: HPMC formed large particles via droplet collision and aggregation, whereas molasses and ammonium nitrate produced more uniform and stable particles mainly through particle coating and layering [18]. Using *Hydrocotyle sibthorpioides* as the raw material, Fu et al. prepared Mg-modified biochar via MgCl_2_ impregnation followed by pyrolysis at 500 °C. The product was then mixed with SA at a mass ratio of 1:4, dropped into CaCl_2_ for crosslinking, and cured at 348 K to form recyclable particles with a diameter of 2–3 mm. The particles exhibited a specific surface area of 266 m^2^/g and abundant mesopores [19]. SA is a natural polysaccharide extracted from marine brown algae and has been increasingly regarded as an eco-friendly adsorbent due to its excellent biocompatibility and extremely low toxicity [20]. At ambient temperature, the G blocks and M blocks in SA can chelate with multivalent metal ions (e.g., Ca^2+^, Fe^3+^, Al^3+^) to form an egg-box structure with tightly linked chains, namely the hydrogel network [21]. On the one hand, this improves the stability and formability of the biochar composite SA material and prevents agglomeration of the adsorbent; on the other hand, Fe^3+^ can act as active sites to enhance the adsorption capacity toward tetracycline pollutants, and synergistically boost the Fenton-like reaction to degrade OTC, thereby improving the overall removal efficiency. To date, few studies have focused on the combined application of biochar composite SA and Fe^3+^ for OTC removal, and the synergistic mechanism underlying their combination remains unclear. This represents the key research gap that the present study aims to address. Therefore, it is attempted to composite biochar with SA gel to prepare composite microspheres with high adsorption efficiency, stable structure and easy recovery, providing a novel solution for the efficient removal of OTC.

In this work, corn stalk was used as the raw material to prepare corn stalk-derived biochar (BC500) via pyrolysis at 500 °C, which was further oxidatively modified by KMnO_4_ to obtain modified biochar (MBC500). Subsequently, BC500 and MBC500 was mixed with SA gel at a certain ratio and crosslinked under the action of ferric ions (Fe^3+^) to fabricate iron-doped biochar/sodium alginate composite microspheres, denoted as BC500-ALF and MBC500-ALF (collectively known as BC/MBC500-ALF). The adsorption performance of BC/MBC500-ALF, with SA gel as the forming and enhancement component, toward OTC in aqueous solution was systematically investigated, with a focus on exploring the synergistic enhancement mechanism of SA gel in the adsorption process. The preparation parameters and operating conditions were optimized, the adsorption type and particle diffusion process were analyzed, and the removal mechanism of OTC was elucidated. This study provides theoretical and technical support for the practical application of iron-doped biochar composite microspheres—with SA gel as the core forming and enhancement component—in the adsorption of OTC from water.

## 2. Results and Discussion

### 2.1. Structural Characterization of Iron-Doped Biochar Composite Sodium Alginate Microspheres

#### 2.1.1. Pore Structure Analysis (SEM, BET)

**Figure 1:**
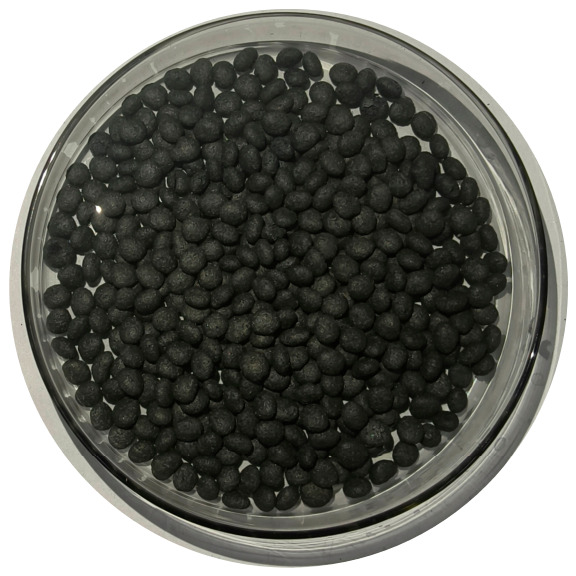
Macromorphology Images of Iron-Doped Biochar/Sodium Alginate Composite Microspheres.

**Figure 2:**
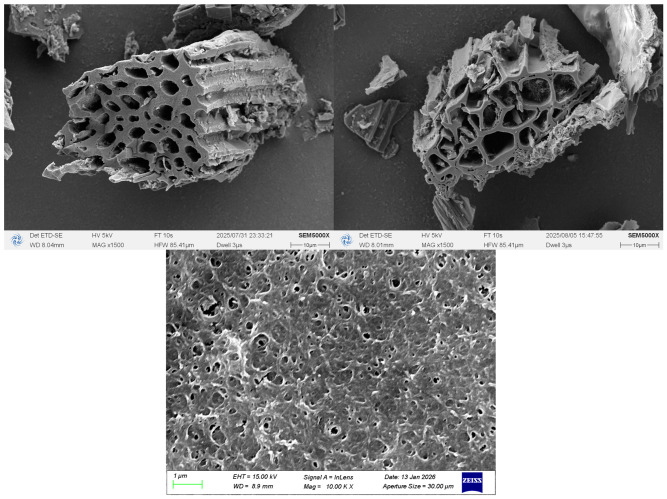
BC500-ALF and MBC500-ALF characterized by SEM.

**Figure 3:**
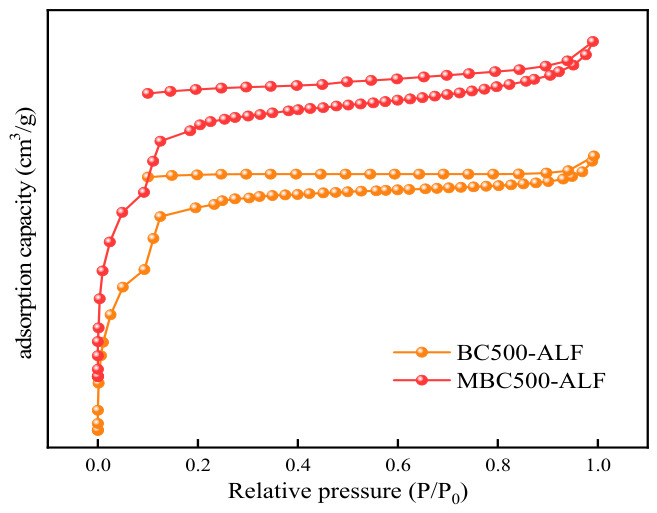
N_2_ adsorption–desorption isotherms.

Figure 1 shows the macroscopic morphology of biochar particles. It can be seen that the iron-doped biochar/sodium alginate composite microspheres are black and spherical, with an average diameter of approximately 2 mm, and the size and shape of each particle are roughly identical. Figure 2 shows the surface morphologies of BC500-ALF and MBC500-ALF characterized by SEM. Figure 3 and Table 1 present the N_2_ adsorption–desorption isotherms and the data table of pore size, specific surface area, and pore volume of the samples calculated via the BET and H-K(Saito-Foley) methods on the basis of N_2_ adsorption–desorption experiments. As illustrated in Figure 2, corn stalk-derived biochar displays a distinct and intact honeycomb-shaped vascular bundle structure. Such a transparent and well-preserved biochar framework provides an ideal substrate for the mass transfer of reactants and exhibits excellent potential for the adsorption of pollutants [22]. The egg-crate-like hydrogel network formed by SA and Fe^3+^ in Figure 2 provides a stable spatial structure for the microspheres.

The N_2_ adsorption–desorption isotherms of both BC500-ALF and MBC500-ALF correspond to Type I isotherms according to the IUPAC 2015 classification, accompanied by a typical Type H4 hysteresis loop [23], indicating that both materials exhibit a hierarchical porous structure dominated by micropores with a small number of slit-shaped mesopores [24].

The adsorption capacity of MBC500-ALF is significantly higher than that of BC500-ALF, confirming that KMnO_4_ oxidative modification effectively improves the specific surface area and micropore volume of the material, providing more active sites for oxytetracycline adsorption. By comparing the graphical and tabular data of BC500-ALF and MBC500-ALF, it can be found that after modification with KMnO_4_, the pore walls of BC500-ALF rupture and collapse, the original regular pore arrangement becomes disordered [25]. The micropore sizes of both materials are highly compatible with oxytetracycline molecules, and the small amount of mesopores serves as mass-transfer channels, conferring distinct advantages for the removal of oxytetracycline from aqueous solutions.

#### 2.1.2. X-Ray Photoelectron Spectroscopy Analysis

**Figure 4:**
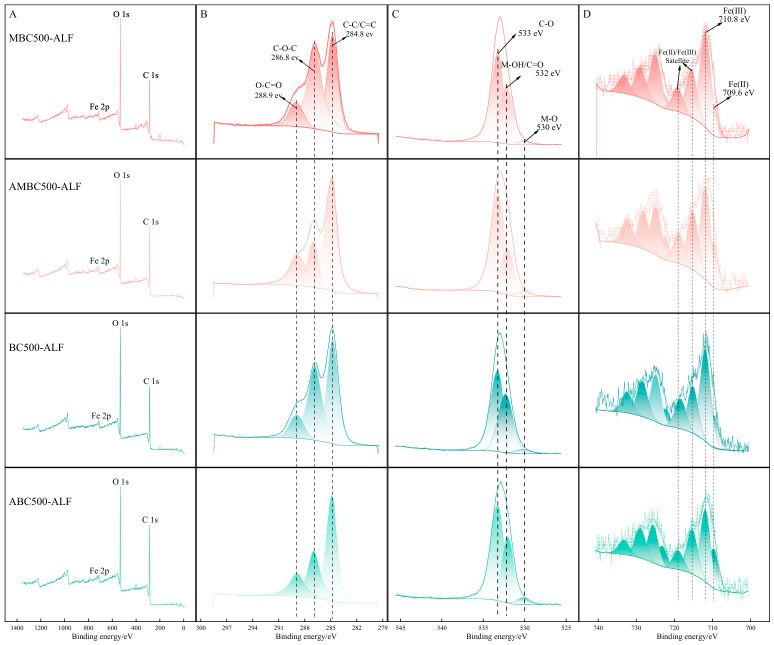
XPS survey and high-resolution spectra of BC500-ALF and MBC500-ALF before and after OTC adsorption. (**A**) XPS survey spectra; (**B**) C 1s spectra; (**C**) O 1s spectra; (**D**) Fe 2p spectra.

Figure 4A shows the surface elemental compositions and chemical functional groups of BC500-ALF and MBC500-ALF before and after OTC adsorption determined by XPS analysis. C 1s, O 1s, and Fe 2p are the main elemental components on the surfaces of BC500-ALF and MBC500-ALF. Figure 4B displays the C 1s spectra of BC500-ALF and MBC500-ALF before and after OTC adsorption, which can be deconvoluted into three peaks at approximately 284.8, 286.8 and 288.9 eV, corresponding to C–C/C=C, C–O–C and O–C=O, respectively [26]. By comparing the peak areas before and after adsorption, a significant decrease in the peak area of C–O–C can be observed. This may be attributed to the fact that oxygen atoms in the C–O–C functional groups act as hydrogen bond acceptors to form hydrogen bonds with active hydrogen atoms in OTC molecules [27], or C–O–C can coordinate with Fe^3+^ present on the biochar surface and further complex OTC molecules through metal-bridging effects, resulting in the consumption of C–O–C functional groups and the decrease in peak area [28].

In the O 1s spectra of BC500-ALF and MBC500-ALF before and after OTC adsorption (Figure 4C), three distinct peaks are observed, assigned to metal oxides (M–O) at approximately 530 eV, metal hydroxides or C=O (M–OH/C=O) at approximately 532 eV, and C–O at approximately 533 eV, respectively [29]. After OTC adsorption, the proportion of M–OH/C=O decreases, while the proportion of M-O increases slightly, indicating that iron and manganese hydroxides may participate in the adsorption process and decompose into M–O attached to the biochar surface.

Figure 4D presents the Fe 2p spectra of BC500-ALF and MBC500-ALF before and after OTC adsorption. The iron oxides on BC500-ALF and MBC500-ALF are mainly composed of Fe(III) and Fe(II). After the reaction, the proportions of Fe(III) on the surfaces of BC500-ALF and MBC500-ALF decrease from 1.26% and 1.54% to 0.94% and 1.15%, respectively. Accordingly, the proportions of Fe(II) increase from 0.4% and 0.34% to 0.48% and 0.43%, respectively. This indicates that redox reactions occur on BC500-ALF and MBC500-ALF during the adsorption reaction [30].

#### 2.1.3. Fourier Transform Infrared Spectroscopy Analysis

**Figure 5:**
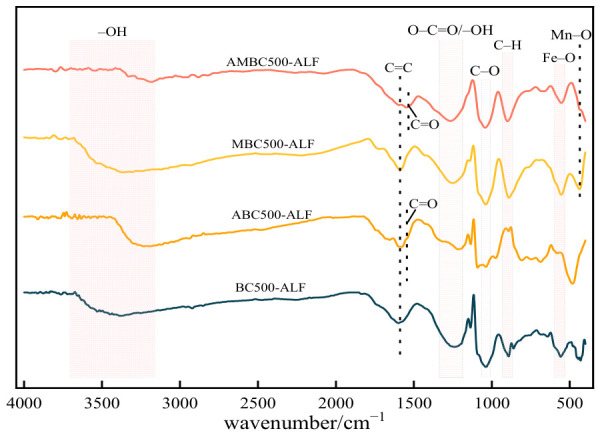
FT-IR Spectra of BC500-ALF and MBC500-ALF.

Figure 5 presents the FT-IR spectra of BC500-ALF and MBC500-ALF before and after OTC adsorption. As shown in the figure, most functional groups of the biochar remained stable before and after oxidative modification. Sharp peaks were observed at approximately 3427–3434, 1628–1632, 1029 and 890–900 cm^−1^, corresponding to −OH stretching vibration [31], C=C stretching vibration [32], C–O functional groups [33] and bending vibration of aromatic C–H bonds [34,35], respectively.

The −OH absorption peaks of BC500-ALF and MBC500-ALF shifted before and after OTC adsorption, which may be a comprehensive result of the enhanced absorption peak induced by C=C–H in the benzene ring and allyl structure of OTC molecules after the biochar adsorbed OTC [36]. The sharp peak at approximately 1224–1266 cm^−1^ was attributed to the bending vibration of −OH in alcohols or phenols, as well as the bending vibration of O–C=O in carboxylic acids [37]. Notably, both BC500-ALF and MBC500-ALF exhibited a new sharp peak at 1621 cm^−1^ after OTC adsorption, which corresponds to C=O stretching vibration [38].

The sharp peak at 435 cm^−1^ was attributed to Mn–O vibration, while the sharp peak at approximately 533 cm^−1^ was assigned to Fe–O vibration, indicating the successful doping of iron [39]. Based on the peak shrinkage at these positions for BC500-ALF and MBC500-ALF before and after OTC adsorption, it can be inferred that Fe–O was involved in the OTC adsorption process. The peak at 435 cm^−1^ confirmed the formation of metal-oxygen bonds at octahedral sites, whereas the peak at 533 cm^−1^ indicated the formation of metal-oxygen bonds at tetrahedral sites of the spinel structure [40]. The intensities of the characteristic Mn–O and Fe–O peaks of BC500-ALF and MBC500-ALF decreased after OTC adsorption, suggesting that manganese oxides and iron oxides may have participated in OTC removal through complexation or been released into the solution [41].

### 2.2. Adsorption Characteristics of Iron-Doped Biochar Composite Sodium Alginate Microspheres for Oxytetracycline

#### 2.2.1. Effects of Initial pH on the Adsorption of OTC by BC500-ALF and MBC500-ALF

**Figure 6:**
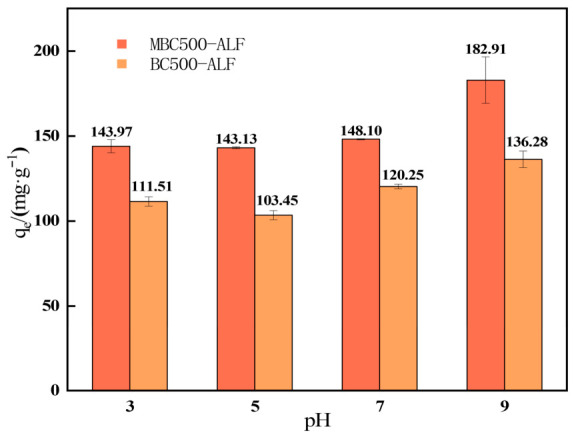
Effect of Initial pH on OTC Adsorption by BC500-ALF and MBC500-ALF.

Variations in solution pH can significantly modify the surface charge properties of biochar (e.g., Zeta potential) as well as the speciation of OTC molecules. By modulating interfacial interactions, including electrostatic interactions and hydrogen bonding, between these two components, the adsorption behavior and efficiency are thereby regulated [42]. Five pH levels (3, 5, 7, 9 and 11) were selected for adsorption experiments, with the results presented in Figure 6.

At pH 11, neither BC500-ALF nor MBC500-ALF could maintain their particulate morphology in the solution. This phenomenon is attributed to the fact that the formation of SA gel depends on cross-linking bonds formed between metal cations and carboxyl groups on the molecular chains. In a strongly alkaline environment, a large number of OH^−^ ions compete with metal cations for carboxyl binding sites or induce the precipitation of metal hydroxides, which ultimately leads to the cleavage of cross-linking bonds and the disruption of the gel structure [43].

It is evident that the adsorption capacities under weakly alkaline conditions were higher than those under acidic and neutral conditions. Specifically, at pH 9, the adsorption capacity of BC500-ALF (136.28 mg/g) was 18% higher than that at pH 3 (111.51 mg/g), while the adsorption capacity of MBC500-ALF (182.91 mg/g) was 21% higher than that at pH 3 (143.97 mg/g).

This observation can be primarily ascribed to the formation of strong hydrogen bonds at pH 9 between the –COO^−^ groups of SA, the oxygen-containing functional groups (e.g., hydroxyl and carboxyl groups) of biochar, and the hydroxyl and amino groups in OTC. Meanwhile, π–π stacking interactions occur between the aromatic ring structures of biochar and the benzene and naphthalene rings in OTC, and this interaction is enhanced by the elevated molecular polarization under alkaline conditions. Furthermore, the –COO^−^ groups of SA form complexes with the hydroxyl and ketone groups of OTC. Particularly in the presence of trace metal cations in the solution, “metal-bridging bonds” can be formed, which further enhances the adsorption performance [44].

**Figure 7:**
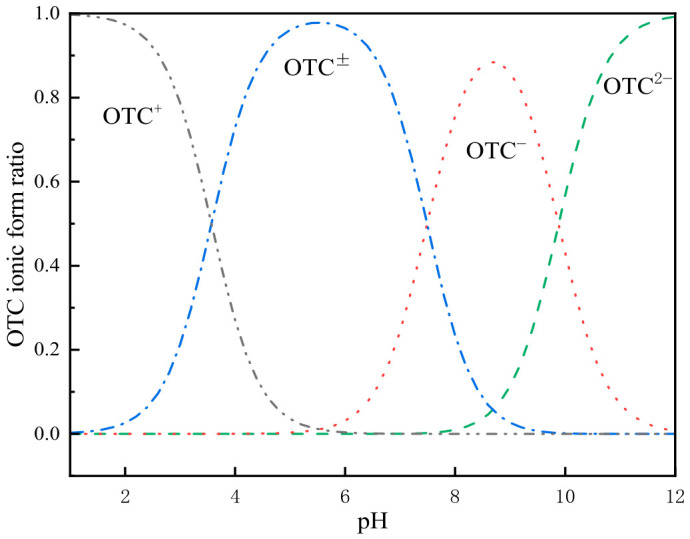
OTC Ion Form Ratio.

On the other hand, the variation in the ionic speciation of OTC with pH is illustrated in Figure 7. OTC has three pKa values, namely 3.23, 7.22 and 8.82. Specifically, OTC predominantly exists as OTC^+^ when pH < 3.23; as OTC^±^/OTC^0^ when 7.22 ≤ pH ≤ 8.82; and as OTC^−^ when pH > 8.82 [45].

Under acidic conditions, electrostatic attraction occurs between the negative charges on the surface of BC/MBC500-ALF and the OTC solution dominated by OTC^+^. However, acidic conditions inhibit the dissociation of carboxyl groups in SA, resulting in weakened hydrogen bonding and complexation interactions between the adsorbent and OTC. With an increase in pH, OTC is transformed into the OTC^±^/OTC^0^ form, where the internal positive and negative charges cancel each other out, leading to a significant reduction in electrostatic interactions with the adsorbent surface. Simultaneously, the adsorption sites inside the biochar are competitively occupied by water molecules or other ions, resulting in a relatively low adsorption capacity.

Under alkaline conditions, OTC^−^ becomes the dominant species in the system. At this pH range, BC/MBC500-ALF exhibits a unique surface charge state: the alkaline nature of biochar enables its surface to retain some positive charge sites, while the carboxyl groups on the SA molecular chains are fully dissociated into –COO^−^ under alkaline conditions, forming a surface structure characterized by the coexistence of positive and negative charge sites.

This specific charge distribution not only provides electrostatic attraction sites for OTC^−^ (via the positive charges on the biochar surface) but also facilitates hydrogen bonding or complexation between –COO^−^ groups and the hydroxyl/amino groups in OTC, thereby significantly improving the adsorption performance of the materials.

#### 2.2.2. Simulation of Kinetic Curves for OTC Adsorption by BC500-ALF and MBC500-ALF

**Figure 8:**
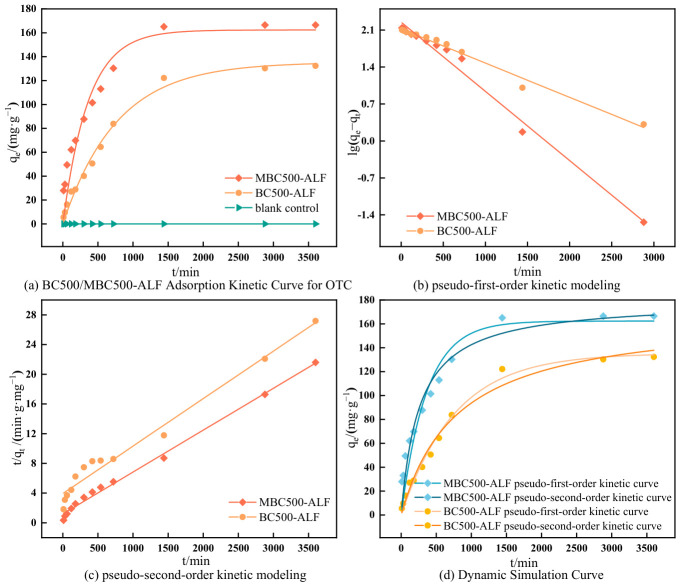
Kinetic Models for OTC Adsorption by BC500-ALF and MBC500-ALF.

To further investigate the adsorption performance of the prepared biochar materials for OTC and its influencing factors, adsorption kinetic simulation studies were conducted on the OTC adsorption processes by BC500-ALF and MBC500-ALF to explore the adsorption mechanism and diffusion mode. Figure 8 shows the fitting curves of the kinetic models for OTC adsorption by BC500-ALF and MBC500-ALF, and the relevant parameters are listed in Table 2.

It can be seen from Figure 8a that the OTC adsorption process by biochar exhibits kinetic characteristics from rapid adsorption to equilibrium. The adsorption capacity increased rapidly in the first 12 h; as time prolonged, the adsorption rate gradually slowed down and eventually reached equilibrium. This is because, in the initial stage of adsorption, the biochar surface has a large number of pore structures and active adsorption sites, and the OTC concentration in the solution is relatively high, resulting in a strong concentration gradient driving force between them [46,47]. Under this condition, OTC molecules can rapidly diffuse and bind to the adsorption sites on the biochar surface, thus showing a rapid increase in adsorption. After 12 h, the number of adsorption sites on the biochar surface decreased, the OTC content in the solution reduced, and the adsorption rate slowed down and tended to equilibrium.

Figure 8b,c show the pseudo-first-order and pseudo-second-order kinetic model simulations for OTC adsorption by BC500-ALF and MBC500-ALF. It can be observed that the experimental data of both biochars have good fitting effects with both the pseudo-first-order and pseudo-second-order kinetic models. As shown in Table 2, the correlation coefficients (*R*^2^) of the pseudo-first-order and pseudo-second-order kinetic models for BC500-ALF and MBC500-ALF are all greater than 0.95, indicating that both adsorption processes are a combination of physical and chemical adsorption [48].

For BC500-ALF, characterization results show it has few oxygen-containing functional groups, an unoptimized pore structure, and limited active sites from iron doping. Thus, its OTC adsorption is dominated by physisorption via van der Waals forces, hydrogen bonding, and pore trapping, with weak chemisorption from iron species. The good agreement between the Q_max_ and experimental data, along with the high fitting accuracy of the pseudo-first-order kinetic model (*R*^2^ = 0.983), confirms physisorption dominance and the supplementary role of iron doping.

For MBC500-ALF, iron doping and KMnO_4_ oxidation exert a strong synergistic effect on the adsorption mechanism. Characterization reveals that KMnO_4_ oxidation introduces abundant oxygen-containing functional groups, which chemically interact with amino and hydroxyl groups in OTC. Iron dopants serve as key active sites, forming stable complexes with OTC through strong coordination. The synergistic modification also optimizes the pore structure, increases specific surface area, and provides more accessible sites for chemisorption; additionally, iron doping may catalyze OTC adsorption and immobilization to enhance chemisorption. The good agreement between the Q_max_ and experimental data, together with the high fitting accuracy of the pseudo-second-order model (*R*^2^ = 0.994), indicates chemisorption dominance, mainly driven by the synergistic effect of iron doping and KMnO_4_ oxidation.

From the fitting data, the adsorption capacity of MBC500-ALF (*q_e_* = 177.30 mg/g) is 25% higher than that of BC500-ALF (*q_e_* = 133.03 mg/g), which demonstrates that KMnO_4_ oxidative modification can significantly improve the OTC adsorption performance of biochar.

#### 2.2.3. Simulation of Adsorption Isotherms for Oxytetracycline by BC500-ALF and MBC500-ALF

**Figure 9:**
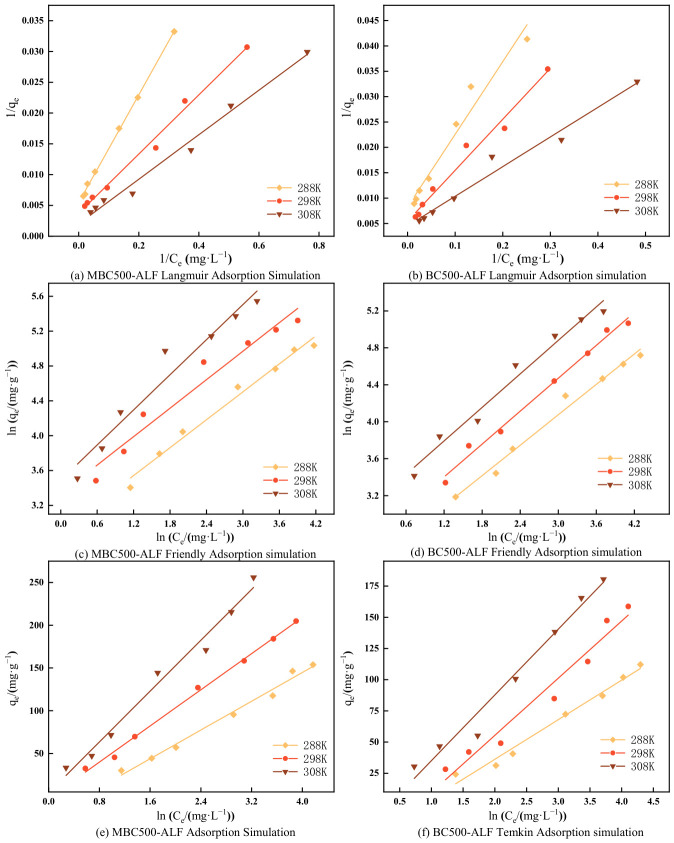
Effect of Temperature on OTC Adsorption by BC500-ALF and MBC500-ALF and Isothermal Adsorption Modeling.

To investigate the effect of ambient temperature on the adsorption of OTC in solution by BC500-ALF and MBC500-ALF, a series of OTC solutions with different initial mass concentrations were selected, and isothermal adsorption model simulation experiments were conducted at 288, 298 and 308 K for each group. The experimental data were analyzed and fitted using three isothermal adsorption models, namely Langmuir, Freundlich, and Temkin, through data simulation and analysis. The fitting results are shown in Figure 9 and Table 3 and Table 4 list the relevant parameters involved in the model fitting.

It can be seen from Figure 9a–d and Table 3 that the *R*^2^ of the Langmuir and Freundlich models for OTC adsorption in solution by BC500-ALF and MBC500-ALF are all above 0.9 (0.944~0.998), indicating that the adsorption process may be a superposition of monolayer adsorption (site binding) and multilayer adsorption (pore filling, intermolecular stacking) [49]. At low concentrations, OTC molecules preferentially bind to the high-activity sites on the biochar surface, dominated by monolayer adsorption. At high concentrations, after the sites are saturated, molecules undergo multi-layer stacking in the pores or on the surface of adsorbed molecules, increasing the proportion of multilayer adsorption. Therefore, the data in the full concentration range fit both models simultaneously. As the temperature increased from 288 K to 308 K, the maximum adsorption capacity of BC500-ALF increased from 120.63 mg/g to 220.75 mg/g, and that of MBC500-ALF increased from 183.15 mg/g to 495.05 mg/g. The adsorption capacity of biochar gradually increased with the increase of adsorption temperature from 288 K to 308 K, from which it can be inferred that the OTC adsorption processes by BC500-ALF and MBC500-ALF are spontaneous endothermic reactions. At 308 K, the maximum adsorption capacity of MBC500-ALF (495.05 mg/g) was 55% higher than that of BC500-ALF (220.75 mg/g), showing a prominent advantage in adsorption performance, which fully demonstrates that KMnO_4_ oxidative modification is an efficient modification strategy.

Analysis of the separation factor (*R_L_*) of the Langmuir model showed that as the system temperature increased from 288 K to 308 K, the OTC removal rate increased, and the corresponding *R_L_* value also increased, indicating that increasing temperature promotes the improvement of adsorption efficiency, which means that the adsorption process is an endothermic process [50]. The *R_L_* values calculated under all temperature conditions fell within the range of 0~1, confirming that the adsorption processes of the two materials have a positive trend at different temperatures [51]. From the analysis of Freundlich model parameters, the adsorption intensity coefficient (n value) is in the range of 1~10, indicating that there is a strong adsorption affinity between OTC molecules and the surfaces of the two materials, and this affinity is sufficient to drive the adsorption process to proceed spontaneously and efficiently, further confirming that the adsorption of OTC by the two biochar materials is a thermodynamically favorable process [52]. The Freundlich adsorption isotherm constant *K_F_* is a temperature-dependent quantity, representing the amount of OTC adsorbed on BC500-ALF and MBC500-ALF [53]. Combined with the physical meaning of *K_F_*, it can be seen that increasing temperature can significantly improve the actual adsorption capacity of the two materials for OTC, which is consistent with the conclusion of “the adsorption process is endothermic” in the previous Langmuir model analysis.

Figure 9e,f and Table 4 show the Temkin models and related parameters for OTC adsorption in solution by BC500-ALF and MBC500-ALF. This model indicates that there is an electrostatic interaction mechanism in the adsorption process [54]. The Temkin model has a better fitting effect on MBC500-ALF (*R*^2^ = 0.979~0.996) than on BC500-ALF (*R*^2^ = 0.954~0.979), which means that with the increase in adsorption capacity, the heat of interaction between the adsorbent and OTC decreases linearly [55]. At different temperatures, the Temkin constant (*K_T_*) of MBC500-ALF is significantly higher than that of BC500-ALF, indicating that the interaction force between OTC and the surface of MBC500-ALF is stronger [56].

In summary, increasing temperature is beneficial to the adsorption of OTC by biochar, and the adsorption process is spontaneous and endothermic.

#### 2.2.4. Simulation of Adsorption Thermodynamic Models for OTC by BC500-ALF and MBC500-ALF

**Figure 10:**
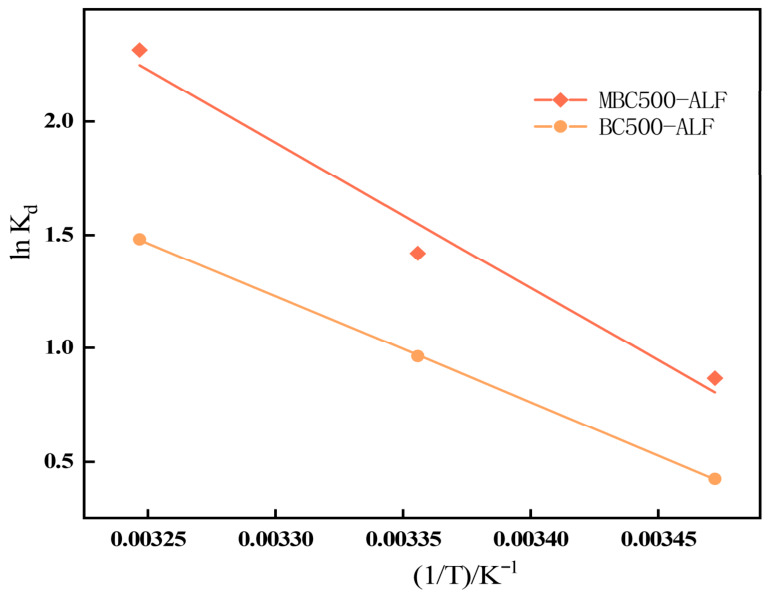
BC500-ALF and MBC500-ALF Adsorption Thermodynamic Relationship Curves for OTC.

To investigate the thermodynamic behavior of OTC adsorption from aqueous solution by BC500-ALF and MBC500-ALF, thermodynamic experiments were carried out at various temperatures. The corresponding thermodynamic parameters were calculated based on the fitting results and parameters of the Langmuir isotherm model. The results are presented in Figure 10 and Table 5.

As shown in Table 5, the Gibbs free energy changes (Δ*G*) of OTC adsorption by BC500-ALF and MBC500-ALF were negative at 288 K, 298 K and 308 K, indicating that the adsorption of OTC onto biochar was a spontaneous process [57]. The absolute value of Δ*G* for BC500-ALF increased from 1.017 kJ/mol at 288 K to 3.794 kJ/mol at 308 K. For the oxidized modified biochar MBC500-ALF, the absolute value of Δ*G* increased from 2.076 kJ/mol at 288 K to 5.924 kJ/mol at 308 K. The increase in the absolute value of Δ*G* with rising temperature demonstrates that the adsorption of OTC by BC500-ALF and MBC500-ALF is spontaneous, and a higher temperature favors a faster adsorption rate and quicker attainment of equilibrium, suggesting better performance of the OTC adsorbents at elevated temperatures [10].

Analysis of the enthalpy change (Δ*H*) shows that Δ*H* values for both biochars were positive, confirming that the adsorption of OTC was endothermic [56], which is consistent with the conclusion from the isotherm analysis that higher temperature promotes the adsorption process. Δ*H* serves as a key indicator to distinguish physisorption and chemisorption: Δ*H* values within 80~200 kJ/mol and 2.1~20.9 kJ/mol correspond to chemisorption and physisorption, respectively [58]. The Δ*H* values of BC500-ALF and MBC500-ALF fell between the ranges for physisorption and chemisorption, indicating a synergistic contribution of both mechanisms during OTC removal by biochar, in agreement with the kinetic analysis.

The entropy changes (Δ*S*) were also positive for both samples. The Δ*S* value of MBC500-ALF was 0.191 J/(mol·K), suggesting increased randomness at the solid–liquid interface and enhanced ordering of adsorption sites during OTC adsorption, which contributes to the spontaneity of the overall process [59].

## 3. Conclusions

BC/MBC500-ALF were successfully fabricated via Fe^3+^ cross-linking, employing corn stalk biochar and KMnO_4_-modified biochar as adsorbent components and SA as an eco-friendly shaping matrix and functional promoter. SA chelated with Fe^3+^ to form a stable egg-box hydrogel network, converting loose powdered biochar into uniform, mechanically robust microspheres. This strategy effectively solved the difficult separation, easy loss, and secondary pollution issues of conventional powdered biochar, significantly improving its engineering applicability. Meanwhile, oxygen-containing functional groups in SA synergized with biochar and iron ions to provide abundant active sites, greatly enhancing adsorption toward OTC.

Batch adsorption experiments indicated that the optimal initial pH was 9. At this condition, the adsorption capacities of BC500-ALF and MBC500-ALF reached 136.28 mg/g and 182.91 mg/g, 18% and 21% higher than those at pH 3, respectively. Kinetic analysis demonstrated that BC500-ALF followed pseudo-first-order kinetics (*R*^2^ = 0.983) dominated by physisorption, while MBC500-ALF fitted pseudo-second-order kinetics (*R*^2^ = 0.994) dominated by chemisorption, with an equilibrium adsorption capacity 25% higher than BC500-ALF.

Adsorption isotherms at 288–308 K were well fitted by Langmuir and Freundlich models. The maximum monolayer adsorption capacities at 308 K were 220.75 mg/g for BC500-ALF and 495.05 mg/g for MBC500-ALF, with the latter 55% higher, verifying the significant enhancement of KMnO_4_ modification. Thermodynamic parameters (ΔG < 0, Δ*H* > 0, Δ*S* > 0) confirmed that the adsorption was spontaneous, endothermic, and accompanied by increased solid–liquid interfacial disorder.

Based on SEM, BET, FT-IR and XPS characterizations combined with kinetic, isotherm and thermodynamic analyses, the adsorption mechanism of OTC onto BC/MBC500-ALF was systematically revealed. The adsorption process follows a continuous pathway: liquid film diffusion, intraparticle diffusion, multi-site interfacial binding and stable immobilization.

Driven by concentration gradient, OTC molecules first diffuse from bulk solution to the microsphere surface, then penetrate into the hierarchical pore network via the egg-box hydrogel network cross-linked by SA and Fe^3+^. KMnO_4_ modification increases specific surface area and pore volume, accelerating mass transfer and exposing more active sites.

At the optimal pH of 9, OTC exists mainly as anions. Strong electrostatic attraction is generated by the amphoteric surface of microspheres. Meanwhile, hydroxyl and carboxyl groups on SA and biochar form hydrogen bonds with OTC. Aromatic domains of biochar produce π–π electron donor–acceptor stacking with conjugated rings of OTC. Fe^3+^ and Mn species act as metal-bridging centers for coordination interaction. In addition, Fe–Mn oxides mediate slight redox reactions to enhance OTC fixation.

Eventually, OTC is firmly anchored on the surface and in pores of microspheres under synergistic interactions to reach equilibrium. SA contributes to both structural stabilization and active site supply. Iron doping and KMnO_4_ modification further optimize surface properties, resulting in superior adsorption capacity of the composite microspheres for OTC.

## 4. Materials and Methods

### 4.1. Reagents and Instruments

Reagents: Oxytetracycline Hydrochloride (OTC, USP grade, Nanjing Dulei Biotechnology Co., Ltd., Nanjing, China), Sodium Alginate (SA, Analytical Reagent, AR), Potassium Permanganate (KMnO_4_, AR), Hydrochloric Acid (HCl, AR), Sodium Hydroxide (NaOH, AR).

Instruments: Electronic Analytical Balance (Model AR2140, Ohaus Instruments Co., Ltd., Shanghai, China), pH Meter (Model DZS-706-B, Leici., Shanghai, China), Electrically Heated Blast Drying Oven (Model DHG, Shanghai Yiheng Scientific Instruments Co., Ltd., Shanghai, China), Muffle Furnace (Model SX3-10-14, Xiangtan Instrument Co., Ltd., Xiangtan, China), Horizontal Constant Temperature Shaker (Model ZWY-200D, Shanghai Zhicheng Analytical Instrument Manufacturing Co., Ltd., Shanghai, China), UV-Vis Spectrophotometer (Model Cary60, Agilent Technologies, Inc., CA, USA), Specific Surface Area Analyzer (Quantachrome Autosorb iQ, FL, USA), Scanning Electron Microscope (SEM 5000X + UltimMax 40e, Guoyi Quantum, Beijing, China), Fourier Transform Infrared Spectrometer (IRTracer 100, Shimadzu, Kyoto, Japan), X-ray Diffraction Analyzer (NEXSA, Thermo Fisher Scientific, MA, USA).

### 4.2. Preparation of Iron-Doped Biochar/Sodium Alginate Composite Microspheres

#### Preparation of Iron-Doped Biochar Composite Microspheres Using Sodium Alginate Gel as the Structuring Matrix

The synthetic procedure employed in this study was mainly adapted from the preparation strategy of biochar-sodium alginate composite microspheres reported by Zou W et al. [60], and further optimized by introducing an additional drying step using a vacuum dryer. A certain amount of corn stalks was washed and dried to a constant weight in an oven at 105 °C. After being crushed by a plant crusher, the stalks were sieved through a 100-mesh sieve to obtain corn stalk powder. Then, 20 g of corn stalk powder was weighed and placed in a ceramic crucible, which was covered to maintain an oxygen-limited environment. The crucible was heated in a muffle furnace at a heating rate of 15 °C/min to 500 °C for pyrolysis for 2 h, yielding corn stalk-derived biochar (BC500).

A 0.5 g/L KMnO_4_ solution was prepared, and the as-prepared BC500 was impregnated at a solid-to-liquid ratio of 1:100. The reaction device was placed in a constant temperature shaker at a rotating speed of 90 r/min for oxidative modification for 12 h. After modification, a circulating water multi-purpose vacuum pump was used to separate the modified biochar from the suspension, which was then washed with deionized water until the pH of the filtrate remained constant. The obtained KMnO_4_-modified biochar was dried to a constant weight in an oven at 80 °C, stored in a sealed bag, and denoted as MBC500.

Approximately 1 g of SA powder was weighed and placed in 50 mL of deionized water, stirred with a magnetic stirrer at a rotating speed of 720 r/min for 1 h, and then cooled for later use. Biochar (BC500 and MBC500) was weighed at a mass ratio of 1:1, dispersed in the prepared 50 mL SA solution, and stirred with a magnetic stirrer at 720 r/min for 4 h to form a homogeneous suspension.

Approximately 3.0 g of anhydrous ferric chloride was weighed and fully dissolved in 300 mL of a solution containing 0.05 mol·L^−1^ polyacrylic acid (PAA) to obtain a PAA double crosslinking curing solution. The suspension was added to a separatory funnel and dropped uniformly into the PAA double crosslinking curing solution. After crosslinking and curing for 12 h, the product was washed with deionized water, dried in a vacuum freeze dryer for 24 h, and the iron-doped biochar composite sodium alginate microspheres were obtained, denoted as BC500-ALF and MBC500-ALF (collectively known as BC/MBC500-ALF).

### 4.3. Characterization and Analytical Method

Microscopic morphology and micro-region elemental composition of biochar samples were characterized using a Quantum SEM 5000X field-emission scanning electron microscope equipped with an UltimMax 40e energy-dispersive X-ray spectrometer (Guoyi Quantum Technology Co., Ltd., Beijing, China). The pore structure, surface morphology, and particle distribution of biochar were clearly visualized.

Prior to measurement, in situ vacuum degassing was conducted on biochar samples with a BSD-660M A3M high-throughput surface area and pore size analyzer (Beshide Instrument Co., Ltd., Beijing, China). Nitrogen adsorption–desorption isotherms were acquired via low-temperature nitrogen physisorption. Correspondingly, the BET specific surface area, pore volume, and pore size distribution parameters were quantitatively calculated.

Surface functional groups of biochar were identified by a Fourier transform infrared spectrometer (IRTracer-100, Shimadzu Corporation, Kyoto, Japan). The species and relative contents of surface functional groups (e.g., hydroxyl, carboxyl, and aromatic groups) were analyzed based on infrared spectra, to clarify the variations in surface chemical structure before and after biochar modification.

X-ray photoelectron spectroscopy tests were performed on a NEXSA X-ray photoelectron spectrometer (Thermo Fisher Scientific, Waltham, MA, USA) for qualitative and semi-quantitative analysis of surface elemental composition, chemical valence states and functional group distributions. The existing chemical forms of C, O and doped elements were precisely characterized, so as to reveal the surface chemical microenvironment and modification mechanism of modified biochar.

### 4.4. Adsorption Characteristic Experiments

#### 4.4.1. Effect of Initial pH on OTC Adsorption by Iron-Doped Biochar/Sodium Alginate Composite Microspheres

An OTC solution with a mass concentration of 50 mg/L was prepared, and 100 mL of the solution was transferred into an Erlenmeyer flask. Subsequently, 0.1 mol/L HCl and 0.1 mol/L NaOH solutions were prepared to adjust the pH of the OTC solution to 3, 5, 7, 9 and 11, respectively. In this procedure, the exact initial concentration of OTC is uncertain. Therefore, the actual OTC concentration was re-measured after pH adjustment, and this measured concentration was used for calculating the adsorption capacity to ensure accuracy and reliability. Then, 20 mg of BC500-ALF and MBC500-ALF were added into each flask, and adsorption was conducted in a constant-temperature oscillator at 25 °C with a rotating speed of 180 r/min for 24 h. After adsorption, the suspension was filtered through a 0.45 µm mixed cellulose ester (MCE) membrane, and the absorbance was measured at 355 nm using a UV-vis spectrophotometer.

For each initial pH value, a blank control group was established simultaneously to eliminate environmental interference and accurately reflect the natural loss of the OTC solution under the corresponding pH condition. Parallel experiments with the same mass of biochar composite were also conducted. Each flask was sampled twice, and each sample was measured five times. The average value was taken as the absorbance of the sample to calculate the equilibrium concentration and adsorption capacity of OTC.

#### 4.4.2. Adsorption Kinetics of OTC by Iron-Doped Biochar/Sodium Alginate Composite Microspheres

An OTC solution with a mass concentration of 50 mg/L was prepared. 100 mL of the solution was transferred into a 150 mL Erlenmeyer flask, and 20 mg of BC500-ALF and MBC500-ALF were added, respectively. The adsorption was carried out in a constant-temperature oscillator at 25 °C with a rotating speed of 180 r/min. Samples were collected at 0, 30, 60, 120, 180, 300, 420, 540, 720, 1440, 2160, 2880 and 3600 min, filtered through a 0.45 μm MCE membrane, and measured at 355 nm using a UV-vis spectrophotometer. Each sample was measured five times, and the average value was used to calculate the solution concentration and adsorption capacity.

A blank control group without biochar and parallel groups with the same mass of biochar were set up. All other conditions remained unchanged, and the above procedures were repeated.

#### 4.4.3. Adsorption Isotherms of OTC by Iron-Doped Biochar/Sodium Alginate Composite Microspheres

Seven groups of OTC solutions with different mass concentrations (10, 15, 20, 40, 60, 80 and 100 mg/L) were prepared. 100 mL of each solution was transferred into a 150 mL Erlenmeyer flask, and 20 mg of BC500-ALF and MBC500-ALF were added, respectively. The mixtures were shaken in a constant-temperature oscillator at 180 r/min for 24 h under 288, 298 and 308 K, respectively. Each sample was collected twice, filtered through a 0.45 μm MCE membrane, and measured five times at 355 nm using a UV–vis spectrophotometer. The average absorbance was adopted to calculate the equilibrium concentration and adsorption capacity.

A biochar-free blank control group and parallel experimental groups with identical biochar dosage were established. All other experimental variables were maintained at a constant level, and the aforementioned experimental operations were conducted in the same manner.

### 4.5. Data Processing

#### 4.5.1. Calculation of Adsorption Capacity

The equilibrium adsorption capacity was calculated by the following equation:

(1) $$q_{e}=\frac{\rho_{0}-\rho_{e}}{m}V$$

where *q_e_* is the equilibrium adsorption capacity of OTC by BC/MBC500-ALF (mg/g), *ρ*_0_ is the initial mass concentration of the contaminant (mg/L), *ρ_e_* is the mass concentration of the contaminant at adsorption equilibrium (mg/L), m is the dosage of BC/MBC500-ALF (g), and *V* is the volume of the solution (L).

#### 4.5.2. Adsorption Kinetic Models

Pseudo-first-order kinetic equation:

(2) $$\ln\left(q_{e}-q_{t}\right)=\ln q_{e}-k_{1}\times t$$

Pseudo-second-order kinetic equation:

(3) $$\frac{t}{q_{t}}=\frac{1}{k_{2}q_{e}^{2}}+\frac{t}{q_{e}}$$

where *q_e_* and *q_t_* are the equilibrium adsorption capacity and the adsorption capacity at time t of OTC by BC/MBC500-ALF, respectively (mg/g); *k*_1_ and *k*_2_ are the adsorption rate constants simulated by the pseudo-first-order and pseudo-second-order kinetic models, respectively; t is the reaction time.

#### 4.5.3. Adsorption Isotherm Models

Common adsorption isotherm models include the Langmuir, Freundlich, and Temkin adsorption isotherm models.

The Langmuir adsorption isotherm model is mainly suitable for monolayer adsorption processes and is expressed as:

(4) $$q_{e}=\frac{K_{L}q_{m}C_{e}}{1+K_{L}C_{e}}$$

(5) $$R_{L}=\frac{1}{1+K_{L}C_{e}}$$

The Freundlich isotherm model is employed to describe multilayer adsorption on heterogeneous sites and is expressed as:

(6) qe=KFCe1n

The Temkin adsorption isotherm model is used to analyze the adsorption behavior between two substances at different temperatures and surface coverage densities, and is expressed as:

(7) $$q_{e}=\frac{R_{1}T}{b}\ln K_{T}+\frac{R_{1}T}{b}\ln C_{e}$$

where *q_e_* is the equilibrium adsorption capacity of OTC by BC/MBC500-ALF (mg/g), *q_m_* is the maximum monolayer adsorption capacity (mg/g), *C_e_* is the equilibrium concentration of OTC in solution (mg/L), *K_L_* is the Langmuir adsorption equilibrium constant (L/mg), *R_L_* is the separation factor, *K_F_* is the Freundlich constant related to adsorption capacity, 1/n is the Freundlich exponent, *R_1_* is the ideal gas constant (8.314 J/(mol·K)), *T* is the thermodynamic temperature (K), b is the Temkin constant (J/mol), and *K_T_* is the Temkin adsorption constant (L/g).

#### 4.5.4. Adsorption Thermodynamic Curve Models

To further investigate the thermodynamic characteristics of BC/MBC500-ALF during the adsorption of OTC solution and the reaction types involved in the adsorption process, the thermodynamic parameters of BC/MBC500-ALF adsorbing OTC were simulated and calculated via the following relevant equations:

(8) $$\Delta G=-R_{1}T\ln K_{d}$$

(9) $$\ln K_{d}=\frac{\Delta S}{R_{1}}-\frac{\Delta H}{R_{1}T}$$

where Δ*G* represents the Gibbs free energy with a unit of kJ/mol; R_1_ is the ideal gas constant (8.314 J/(mol·K)); *T* is the thermodynamic temperature in K; *K_d_* refers to the adsorption equilibrium constant; Δ*S* stands for the standard entropy change with a unit of J/(mol·K); Δ*H* represents the standard enthalpy change with a unit of kJ/mol.

## Figures and Tables

**Table 1:** Comparison of Specific Surface Area, Pore Size, and Pore Volume for BC500-ALF and MBC500-ALF.

Biochar	BC500-ALF	MBC500-ALF
Specific surface area/(m^2^·g^−1^)	134.36	144.95
Pore size/nm	1.3758	1.4367
Pore volume/(cm^3^·g^−1^)	0.0203	0.0249

**Table 2:** Kinetic Model Parameters for OTC Adsorption by BC500-ALF and MBC500-ALF.

Biochar	Pseudo-First-Order Kinetic Model			Pseudo-Second-Order Kinetic		
	*q*_e_	*k*_1_	*R* ^2^	*q*_e_	*k*_2_	*R* ^2^
BC500-ALF	133.03	−6.486 × 10^−4^	0.983	156.49	1.04 × 10^−5^	0.973
MBC500-ALF	177.88	−1.31 × 10^−3^	0.984	177.30	2.66 × 10^−5^	0.994

**Table 3:** Fitting Parameters for Langmuir and Freundlich Adsorption Isotherms of OTC for BC500-ALF and MBC500-ALF.

Biochar	T/K	Langmuir Model				Freundlich Model		
		*K*_L_ (L·mg^−1^)	*q*_m_/(mg·g^−1^)	*R* ^2^	*R*_L_	*K_F_*/((mg·g^−1^) (L·mg^−1^)^1/n^)	*n*	*R* ^2^
BC500-ALF	288	0.058	120.63	0.954	0.190~0.812	11.344	1.822	0.982
	298	0.054	182.82	0.976	0.233~0.844	14.716	1.683	0.988
	308	0.078	220.75	0.971	0.239~0.861	21.479	1.654	0.972
MBC500-ALF	288	0.062	183.15	0.998	0.199~0.836	18.209	1.872	0.944
	298	0.044	268.82	0.990	0.207~0.879	28.103	1.835	0.981
	308	0.056	495.05	0.986	0.414~0.932	32.811	1.489	0.947

**Table 4:** Fitting Parameters for the Temkin Adsorption Isotherm of OTC for BC500-ALF and MBC500-ALF.

Biochar	T/K	Temkin Model		
		*K*_T_/(L·g^−1^)	*b*/(KJ·mol^−1^)	*R* ^2^
BC500-ALF	288	0.717	45.465	0.979
	298	0.453	54.068	0.954
	308	0.424	80.722	0.972
MBC500-ALF	288	1.056	32.219	0.985
	298	0.956	46.854	0.996
	308	0.572	60.845	0.979

**Table 5:** Thermodynamic Parameters of OTC Adsorption by BC500-ALF and MBC500-ALF.

Biochar	Δ*G*/(kJ/mol)			ΔH (kJ/mol)	ΔS (J/(K·mol))
	288	298	308		
BC500-ALF	−1.017	−2.387	−3.794	38.962	0.139
MBC500-ALF	−2.076	−3.519	−5.924	53.172	0.191

## Data Availability

The data presented in this study are available on request from the corresponding author. The data are not publicly available due to ongoing studies using a part of the data.
